# Association of vitamins B1 and B2 intake with early-onset sarcopenia in the general adult population of the US: a cross-sectional study of NHANES data from 2011 to 2018

**DOI:** 10.3389/fnut.2024.1369331

**Published:** 2024-03-08

**Authors:** Sha Yang, Zhenyu Dong, Jiaqi Zhao, Lijia Yuan, Yao Xiao, Xing Luo, Zhuyang Zhao, Xia Kang, Kanglai Tang, Ming Chen, Liu Feng

**Affiliations:** ^1^Department of Clinical Laboratory Medicine, Southwest Hospital, Third Military Medical University, Chongqing, China; ^2^Department of Orthopeadics, Sports Medicine Center, Key Laboratory of Sports Injury Repair and Reconstruction, Southwest Hospital, Third Military Medical University, Chongqing, China; ^3^Department of Orthopedic Surgery, Southwest Hospital, Third Military Medical University, Chongqing, China; ^4^Emergency Department, Southwest Hospital, Third Military Medical University, Chongqing, China; ^5^Pancreatic Injury and Repair Key Laboratory of Sichuan Province, The General Hospital of Western Theater Command, Chengdu, Sichuan, China

**Keywords:** early-onset sarcopenia, vitamin B1, vitamin B2, NHANES, weighted multiple logistic regression, RCS

## Abstract

**Background:**

Early-onset sarcopenia refers to the progressive loss of muscle mass and function that occurs at an early age. This condition perpetuates the vicious cycle of muscle loss and is associated with adverse outcomes. It is important to identify the contributing factors for early intervention and prevention. While diet is known to impact muscle mass, the association of B vitamins with early-onset sarcopenia remains unexplored.

**Objectives:**

To investigate the association of B vitamins intake with early-onset sarcopenia risk in a cross-sectional study.

**Methods:**

We conducted data analysis on a total of 8,711 participants aged between 20 and 59 years who took part in the National Health and Nutrition Examination Survey (NHANES) from 2011 to 2018. Early-onset sarcopenia was defined as a SMI measured by DXA that was one standard deviation below the sex-specific mean of the reference population. B vitamins intake (B1, B2, B3, B6, B9, and B12) was assessed by 24-h dietary recall. We used weighted multiple logistic regression and RCS models to estimate the OR and 95% CI of sarcopenia by B vitamins intake, adjusting for demographic, physical, lifestyle, comorbidities, and nutritional covariates.

**Results:**

Higher intake of vitamin B1 was associated with a 22% lower sarcopenia risk (OR = 0.78, CI = 0.63–0.97, *p* = 0.022), and higher intake of vitamin B2 with a 16% lower risk (OR = 0.84, CI = 0.74–0.97, *p* = 0.012) in both genders. Gender-specific analyses showed a 28% reduction in sarcopenia risk among males with each additional mg of vitamin B1 intake (OR = 0.72, CI = 0.52–0.97, *p* = 0.038), and a 26% decrease among females with each additional mg of vitamin B2 intake (OR = 0.74, CI = 0.57–0.96, *p* = 0.021). No significant differences were found between vitamin B2 and males, or between vitamin B1 and females. The RCS model suggested a nonlinear relationship between vitamin B2 intake and sarcopenia risk (*P*_Overall_ = 0.001, *P*_Nonlinear_ = 0.033), with a plateau effect above 3 mg/d.

**Conclusion:**

Higher intake of vitamin B1 and B2 may lower the risk of early-onset sarcopenia, with gender differences. This suggests the potential of nutritional intervention by increasing these vitamins intake through diet and supplements. Further research is warranted to elucidate the mechanisms and design targeted interventions.

## Introduction

Sarcopenia is a disorder that affects the skeletal muscle in a complex and systemic way, leading to a gradual decline in muscle mass and function (1). Its ramifications cascade into a myriad of secondary consequences, precipitating falls, disability, prolonged bedrest, compromised self-sufficiency, and psychological distress (2, 3). In addition, sarcopenia often coexists with a variety of chronic diseases, including congestive heart failure, chronic obstructive pulmonary disease, neurological disorders, diabetes, cancer cachexia, malnutrition and sarcopenic obesity, and these conditions show complex interactions (1, 4–8). This interplay of diseases creates a vicious circle that accelerates disease progression and reduces the quality of life of those affected, contributing significantly to mortality and imposing a substantial social and financial burden on public health (9–13). Sarcopenia is considered a special form of senescence, but recent studies have found that some patients start to lose muscle mass in middle age or even youth (14–16). This type of sarcopenia that progresses in early-life is called early-onset sarcopenia (17–19). Early-onset sarcopenia has worse clinical manifestations and prognosis than ordinary sarcopenia (20). For patients with early-onset sarcopenia, a special type of high-risk population, prevention and intervention measures may effectively delay the progression of the disease and reduce the heavy burden on patients and the medical system (20). Despite its clinical and research importance, the current research on early-onset sarcopenia is still at a preliminary stage, constrained by unclear diagnostic standards and insufficient epidemiological data.

B vitamins are water-soluble compounds that play an important role in cell metabolism. Although abundant in food, these nutrients are vulnerable to loss during the cooking process. Moreover, nutrients can be easily consumed in significant amounts as part of the body’s metabolic processes (21, 22). Marginal deficiency of B vitamins is prevalent in developed societies, particularly among individuals adhering to a “modern Western diet” characterized by excessive cooking and processed foods (23–27). This marginal insufficiency may not exhibit typical symptoms but can have long-term implications for health (28, 29).

Various interrelated factors contribute to the development of early-onset sarcopenia, including inflammation, oxidative stress, dysfunction of energy metabolism, neurodegenerative changes, endocrine changes, impaired energy intake and absorption, and physical inactivity (2, 30–33). Vitamins are important for muscle homeostasis, and the association between vitamin D deficiency and the risk of sarcopenia has been widely reported (34–38). However, few studies have investigated the role of B vitamins in maintaining muscle mass and function. Since B vitamins are directly or indirectly involved in a variety of biological processes, including energy and protein metabolism, maintenance of neurological function, and even relatively short-term deficiencies may accumulate detrimental effects, their contribution to the complex etiology of sarcopenia may be significant and may vary with its stage. Recent studies have provided some evidence: for example, researchers have found that elevated homocysteine levels in older adults may be associated with low muscle mass and poor physical function, which could result from a deficiency of vitamins B6, B9, and B12, which facilitate homocysteine breakdown in the body (39–43). Similarly, in some small-sample retrospective experiments, sarcopenic elderly people had lower intakes of vitamins B6 and B9 than non-sarcopenic elderly people (44–46). Another prospective study involving 403 subjects inferred that the prevalence of sarcopenia was as high as 31.6% in older adults with serum B12 intake less than 400 pg./mL (47). However, in another small randomized controlled trial, it was observed that the supplementation of vitamin B12 and folic acid did not yield a significant delay in the decline of energy levels and grip strength among an older population. Despite growing interest, studies on B vitamins and sarcopenia are hindered by limitations such as small sample sizes and inconsistent findings, leaving significant gaps in understanding. Early prevention and intervention are vital due to the prolonged muscle mass decline in early-onset sarcopenia. Exploring the relationship between B vitamins and early-onset sarcopenia is expected to provide preliminary evidence for B vitamins as a key, feasible, and affordable intervention. Additionally, beyond B vitamins investigating lifestyle factors like socioeconomic status and physical activity levels is also essential. Understanding their interplay with muscle health could offer valuable insights for comprehensive preventive strategies.

This analysis aims to investigate the potential relationship between B vitamins intake and early-onset sarcopenia using data from The National Health and Nutrition Examination Survey (NHANES) 2011–2018.

## Methods

### Experimental design and bias control

NHANES, led by the CDC’s National Center for Health Statistics, assesses the US population’s health and nutrition using a multistage sampling method for broad representation. It employs complex stratification to oversample specific groups and assigns sample weights to ensure national representativeness, considering sampling probabilities and non-response. Trained personnel gather data through interviews, questionnaires, physical exams, and lab tests, following standard protocols. Participants receive compensation and reports, enhancing compliance. Non-response is minimal. All variables, including B vitamin levels, are compared by gender and early-onset sarcopenia status. Data are weighted based on NHANES’ stratification, then modeled to explore B vitamin associations with early-onset sarcopenia.

### Study population

**Figure 1:**
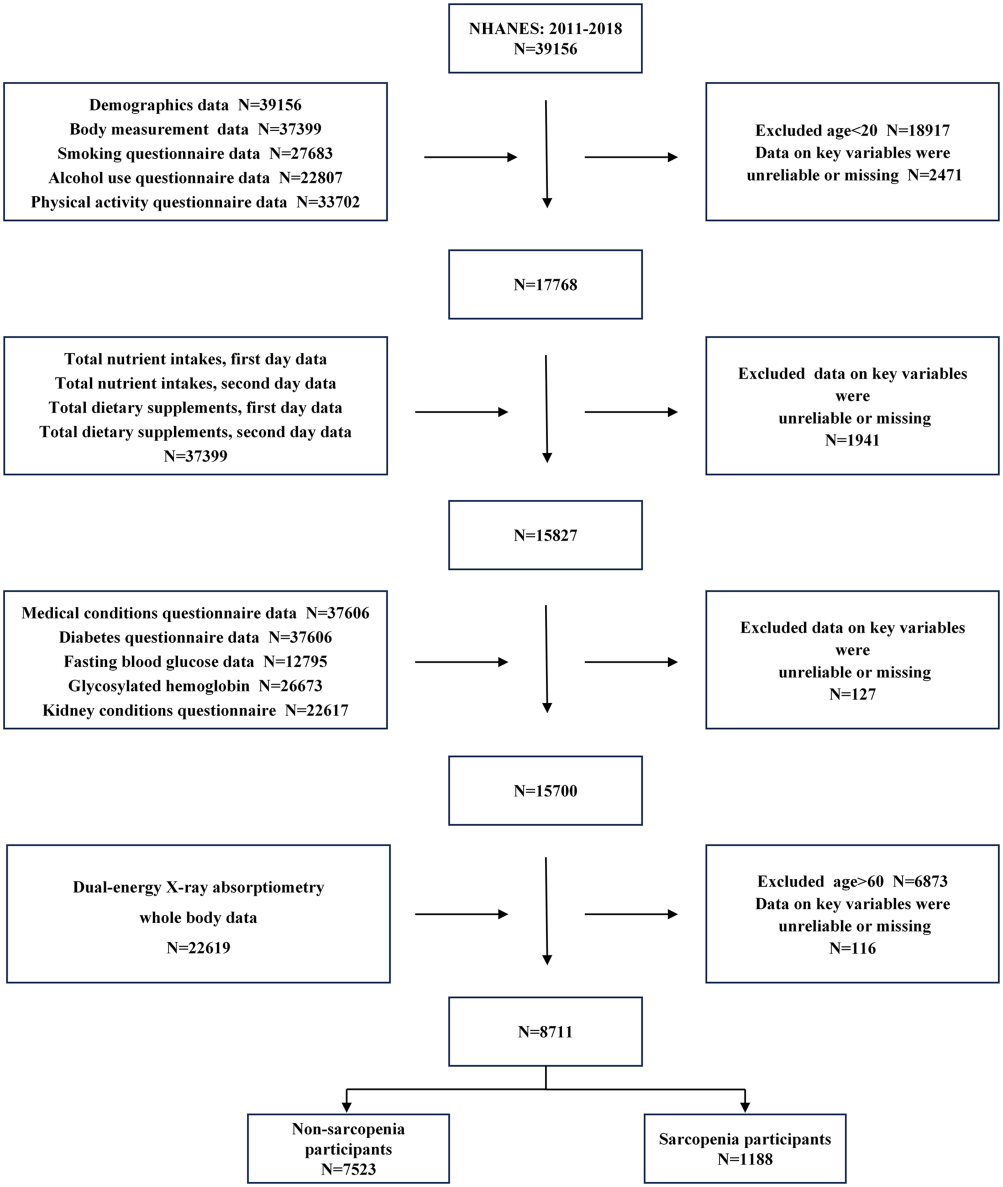
Flowchart for the inclusion of study participants.

We used data from NHANES 2011–2018 across four cycles, with 39,156 screened participants. We merged the data modules by the unique ID of each participant, and only included valid data as classified by the testers. We excluded invalid, missing, or refused data. In total, 8,711 participants were included in the study (Figure 1).

### Demographic and physical examination data

Demographic data were obtained through the administration of questionnaires, with a primary focus on variables such as gender, age, race, and socioeconomic status. Poverty was assessed using the family income ratio (PIR) relative to the poverty threshold, with participants below a PIR of 1.3 categorized as economically disadvantaged. Given the impact of nutritional status on sarcopenia, Body Mass Index (BMI) was included in studies as one of the measures of obesity. BMI is a person’s weight in kilograms divided by height squared. However, it was often considered insufficient to include BMI alone. Therefore, the weight-adjusted waist index (WWI) was introduced, which is calculated by dividing waist circumference (WC) by the square root of weight. WWI is a new type of adiposity index that standardizes WC for weight, reflecting abdominal fat distribution and body shape. Unlike WC, WWI is not influenced by body mass index (BMI), which is a measure of general adiposity that does not take body composition into account.

### Smoking status

Participants were categorized into three groups: non-smokers, current smokers, and ex-smokers. Non-smokers had consumed fewer than 100 cigarettes in their lifetime. Current smokers were actively smoking, while ex-smokers had refrained from smoking for over 3 months and were consuming less than one cigarette per day.

### Alcohol usage status

Participants were divided into non-drinkers, slight to moderate drinkers, and heavy drinkers. Non-drinkers had consumed 12 drinks or fewer in their lifetime. Slight to moderate drinkers were men with a daily intake of less than two drinks and women with a daily intake of less than one drink. Heavy drinkers were identified as men consuming more than two drinks daily and women consuming more than one drink daily.

### Physical activity status

Participants were grouped into inactive, moderately active, and highly active based on their physical activity. Physical activity included work-related or exercise-related energy expenditure. The inactive group did less than 150 min of moderate or higher-intensity activity or less than 75 min of high-intensity activity per week. The moderately active group did 150 to 300 min of moderate or higher-intensity activity or 75 to 150 min of high-intensity activity per week. The highly active group did more than 300 min of moderate or higher-intensity activity or more than 150 min of high-intensity activity per week.

### Other conditions potentially leading to clinical manifestations of sarcopenia

We adjusted for chronic comorbidities that may cause muscle loss, such as heart failure, lung disease, diabetes, cancer, kidney disease, arthritis, and anemia. These diseases can mimic or worsen sarcopenia, a condition of low muscle mass and strength. NHANES Medical Conditions data is used to get self-reported disease information from the participants. We diagnosed diabetes based on the Diabetes questionnaire and lab tests (fasting blood glucose >7 mmol/L or hemoglobin A1c >6.5%). We established criteria for renal insufficiency utilizing the Kidney Conditions questionnaire.

### Intake of B vitamins

Two 24-h recall interviews were used to collect all nutritional data, such as B vitamins, protein, carbohydrate, fat, and calories intake. The types and amounts of food, beverages, and dietary supplements that the participants had in the last 24-h were queried. The automated multiple pass method (AMPM) system was employed by experienced interviewers to conduct the interviews. AMPM is a computer-assisted interview system that follows a five-step, multiple-pass approach with standardized probes, intended to estimate current dietary intake and reduce underreporting. Three-dimensional food models and the USDA Food Model Booklet were utilized to create these standardized probes, which aimed to more precisely estimate the nutrient content of the food, beverages, and dietary supplements that the participants ingested.

### Diagnosis of early-onset sarcopenia

Skeletal muscle mass index (SMI) was the measure of muscle quality. SMI is the appendicular lean mass (without minerals and fat) over height squared. We evaluated body composition using dual-energy X-ray absorptiometry (DXA) in non-pregnant subjects aged 8–59 years. We adopted Janssen’s method and calculated the mean and standard deviation of SMI for men and women aged 20–40 years (48). Early-onset sarcopenia is defined as SMI below one standard deviation of the mean. The cut-off values were 7.44 kg/m^2^ for men and 5.57 kg/m^2^ for women, which was similar to the previous results (49, 50). We found 1,188 sarcopenia patients and 7,523 non-sarcopenia participants. The male cohort consisted of 617 participants with sarcopenia and 3,776 without, while the female cohort comprised 571 participants with sarcopenia and 3,747 without.

### Statistical and analysis

Sample weights were appropriately assigned according to NHANES guidelines, merging samples from different survey cycles. Categorical variables between sarcopenic and non-sarcopenic subjects were compared using the chi-squared test with Rao & Scott’s second-order correction, and continuous variables using the Wilcoxon rank-sum test. Continuous variables were expressed as mean (±SD) or P50 (P25, P75), while categorical variables as unweighted *n* (%). The study utilized multivariable logistic regression and restricted cubic splines to explore B vitamin associations with early-onset sarcopenia, employing four logistic regression models and one restricted cubic splines (RCS) model, stratified by sex for reliable outcomes. Logistic regression also assessed dose-effect trends of B vitamin intake, transformed into ordinal variables, adjusting for potential confounders. Association was estimated using odds ratios (ORs) and 95% confidence intervals (CIs), assessing non-linearity with RCS. Analysis was conducted using R version 4.3.1 and RStudio version 2023.06.2 + 561 Chrome.

## Results

### Baseline characteristics

We analyzed and presented each gender separately in this study due to significant differences in BMI, WWI, SMI, and B vitamins intake. Table 1 showed the nutritional data on dietary sources. Baseline data for the overall population were shown in Supplementary Table S1. The male cohort had 3,776 non-sarcopenia and 617 sarcopenia participants, while the female cohort had 3,747 non-sarcopenia and 571 sarcopenia participants. Male sarcopenia participants were significantly younger (37 ± 13) than non-sarcopenia participants (39 ± 11) (*p* = 0.005), but no difference in age was observed among females. Both genders showed marked differences in ethnicity-wise. Non-Hispanic Asian and White sarcopenia participants had a higher proportion than non-Hispanic African American and Hispanic participants (*p* < 0.001). Male sarcopenia participants had a higher percentage of low household income (27%) than non-sarcopenia participants (21%) (*p* = 0.015), but no difference in income was observed among females. Both genders had lower BMI and WWI values in sarcopenia participants (*p* < 0.001). No lifestyle factors (smoking, alcohol and physical activity) differed between sarcopenia and non-sarcopenia participants. Both genders had higher prevalence of chronic lung diseases and diabetes, but lower prevalence of gout in sarcopenia participants.

**Table 1:** Baseline characteristics of participants by gender.

Characteristic	Male data			Female data		
	Non-sarcopenia participants (*N* = 3,776)	Sarcopenia participants (*N* = 617)	*p*	Non-sarcopenia participants (*N* = 3,747)	Sarcopenia participants (*N* = 571)	*p*
**Age**	39 (±11)	37 (±13)	**0.005**	40 (±12)	39 (±13)	0.5
**Race**			**<0.001**			**<0.001**
Hispanic	937 (18%)	96 (11%)		971 (16%)	111 (13%)	
Non-Hispanic White	1,369 (62%)	263 (69%)		1,392 (64%)	220 (67%)	
Non-Hispanic Black	828 (11%)	61 (5.3%)		844 (12%)	31 (3.0%)	
Non-Hispanic Asian	473 (4.8%)	170 (10%)		379 (4.2%)	191 (14%)	
Other Race	169 (3.6%)	27 (4.2%)		161 (3.8%)	18 (3.1%)	
**PIR status**			**0.015**			0.6
High income households	2,643 (79%)	401 (73%)		2,505 (76%)	404 (77%)	
Low income households	1,133 (21%)	216 (27%)		1,242 (24%)	167 (23%)	
**BMI**	29.5 (±5.4)	22.1 (±2.7)	**<0.001**	30 (±7)	21 (±2)	**<0.001**
**WWI**	10.65 (±0.71)	10.36 (±0.68)	**<0.001**	11.02 (±0.80)	10.64 (±0.66)	**<0.001**
**Alcohol usage status**			0.3			0.12
Non-drinkers	286 (6.3%)	73 (8.1%)		585 (11%)	116 (15%)	
slight to moderate drinkers	3,108 (82%)	486 (79%)		2,874 (78%)	411 (76%)	
Heavy drinkers	382 (11%)	58 (13%)		288 (10%)	44 (8.9%)	
**Smoking status**			0.12			0.5
Non-smokers	2,032 (55%)	320 (47%)		2,503 (64%)	393 (62%)	
Ex-smokers	78 (2.0%)	7 (2.4%)		63 (2.1%)	6 (1.4%)	
Current smokers	1,666 (43%)	290 (51%)		1,181 (34%)	172 (37%)	
**Physical activity status**			0.12			0.6
Inactive group	1,545 (40%)	291 (44%)		2,080 (55%)	349 (58%)	
Moderately active group	296 (8.2%)	60 (10%)		333 (9.8%)	51 (8.9%)	
Highly active group	1,935 (51%)	266 (45%)		1,334 (35%)	171 (33%)	
**Malignancies or Cancers**			0.6			0.2
No	3,685 (97%)	599 (96%)		3,548 (94%)	540 (91%)	
Yes	91 (3.4%)	18 (4.0%)		199 (6.2%)	31 (9.1%)	
**Chronic lung diseases**			**0.041**			**0.035**
No	3,671 (96%)	589 (94%)		3,532 (93%)	530 (89%)	
Yes	105 (3.7%)	28 (5.6%)		215 (6.5%)	41 (11%)	
**Diabetes**			**0.015**			**<0.001**
No	3,467 (92%)	555 (91%)		3,447 (92%)	564 (90%)	
Yes	309 (8.1%)	62 (9.5%)		300 (7.9%)	51 (9.6%)	
**Congestive heart failure**			0.4			0.051
No	3,738 (99%)	612 (100%)		3,713 (99%)	568 (100%)	
Yes	38 (0.6%)	5 (0.4%)		34 (0.9%)	3 (0.3%)	
**Anemia**			0.2			0.8
No	3,759 (100%)	611 (100%)		3,501 (94%)	545 (95%)	
Yes	17 (0.3%)	6 (0.5%)		246 (5.8%)	26 (5.4%)	
**Arthritis**			0.6			>0.9
No	3,373 (89%)	559 (88%)		3,108 (83%)	487 (83%)	
Yes	403 (11%)	58 (12%)		639 (17%)	84 (17%)	
**Gout**			**0.041**			**0.033**
No	3,641 (96%)	605 (99%)		3,693 (99%)	567 (100%)	
Yes	135 (3.6%)	12 (1.4%)		54 (1.4%)	4 (0.4%)	
**Stroke**			0.6			0.6
No	3,728 (99%)	611 (99%)		3,689 (99%)	567 (99%)	
Yes	48 (0.9%)	6 (0.7%)		58 (1.2%)	4 (0.8%)	
**Kidney insufficiency**			0.1			0.5
No	3,725 (99%)	601 (98%)		3,676 (98%)	557 (97%)	
Yes	51 (1.3%)	16 (2.4%)		71 (1.9%)	14 (2.5%)	
**Dietary energy (kcal)**	2,419 (1,898, 3,009)	2,332 (1,821, 2,934)	0.1	1,777 (1,416, 2,195)	1,656 (1,297, 2,123)	**0.014**
**Dietary protein (g)**	94 (72, 120)	88 (67, 111)	**0.003**	69 (54, 87)	64 (47, 81)	**<0.001**
**Dietary carbohydrate (g)**	276 (209, 353)	277 (203, 359)	0.8	212 (160, 267)	207 (155, 266)	0.4
**Dietary fat (g)**	91 (67, 120)	86 (64, 114)	0.058	69 (51, 89)	63 (47, 83)	**<0.001**
**Dietary vitamin B1 (mg)**	1.75 (1.30, 2.28)	1.72 (1.26, 2.23)	**0.034**	1.30 (0.97, 1.70)	1.27 (0.90, 1.66)	**0.044**
**Dietary vitamin B2 (mg)**	2.25 (1.66, 3.03)	2.11 (1.62, 2.89)	**0.036**	1.74 (1.32, 2.31)	1.69 (1.25, 2.11)	**0.009**
**Dietary vitamin B3 (mg)**	30 (23, 39)	28 (21, 37)	**0.01**	21 (16, 27)	20 (15, 26)	**0.044**
**Dietary vitamin B6 (mg)**	2.30 (1.65, 3.05)	2.12 (1.60, 2.97)	0.053	1.63 (1.19, 2.20)	1.61 (1.15, 2.17)	0.4
**Dietary folate (μg)**	165 (100, 266)	168 (112, 278)	0.2	122 (71, 198)	116 (58, 202)	0.4
**Dietary folic acid (μg)**	534 (384, 730)	553 (379, 794)	0.6	410 (291, 571)	409 (284, 577)	0.9
**Dietary vitamin B12 (μg)**	4.9 (3.1, 7.4)	4.7 (3.2, 7.1)	0.4	3.33 (2.20, 5.05)	3.22 (2.13, 4.67)	0.3
**Dietary vitamin B1 (tertiles)**			0.053			0.1
Low intake level	1,248 (33%)	216 (36%)		1,245 (34%)	193 (33%)	
Moderate intake level	1,263 (34%)	201 (32%)		1,253 (32%)	188 (35%)	
High intake level	1,265 (33%)	200 (32%)		1,249 (34%)	190 (32%)	
**Dietary vitamin B2 (tertiles)**			0.3			**0.027**
Low intake level	1,246 (28%)	221 (29%)		1,257 (33%)	182 (36%)	
Moderate intake level	1,251 (34%)	212 (37%)		1,229 (27%)	212 (32%)	
High intake level	1,279 (38%)	184 (34%)		1,261 (40%)	177 (32%)	
**SMI**	9.00 (8.31, 9.84)	7.01 (6.64, 7.26)	**<0.001**	6.96 (6.31, 7.98)	5.26 (5.02, 5.42)	**<0.001**

Age, BMI, and WWI exhibited Gaussian distribution (mean ± SD); nutritional variables and SMI deviated (IQR: P50 (P25, P75)); categorical variables were denoted as unweighted *n* (weighted %). Categorical and continuous variables were compared via chi-squared and Wilcoxon rank-sum tests with Rao & Scott’s correction for complex surveys. Statistical differences (*p* < 0.05) were highlighted in bold.

Female sarcopenia participants consumed less total energy [1,656 (1,297, 2,123) kcal, *p* = 0.014] and fat [63 (47, 83) g, *p* < 0.001] from dietary sources than non-sarcopenia participants [1777 (1,416, 2,195) kcal for total energy and 69 (51, 89) g for fat, respectively]. But, there were no differences in total energy and fat between male sarcopenia participants and male non-sarcopenia participants. And both male and female sarcopenia participants had lower protein intake from the sum of dietary sources and dietary supplements than their respective controls (*p* = 0.003 and *p* < 0.001, respectively). Total carbohydrate intake did not differ significantly. Among the B vitamins from dietary sources, vitamin B1, B2, and B3 showed significant differences in both male and female groups, with sarcopenia participants having lower intakes than non-sarcopenia participants (*p* < 0.05). No significant differences were observed for vitamin B6, folic acid, folate, and vitamin B12.

### Association analysis of B vitamins and the risk of sarcopenia

**Figure 2:**
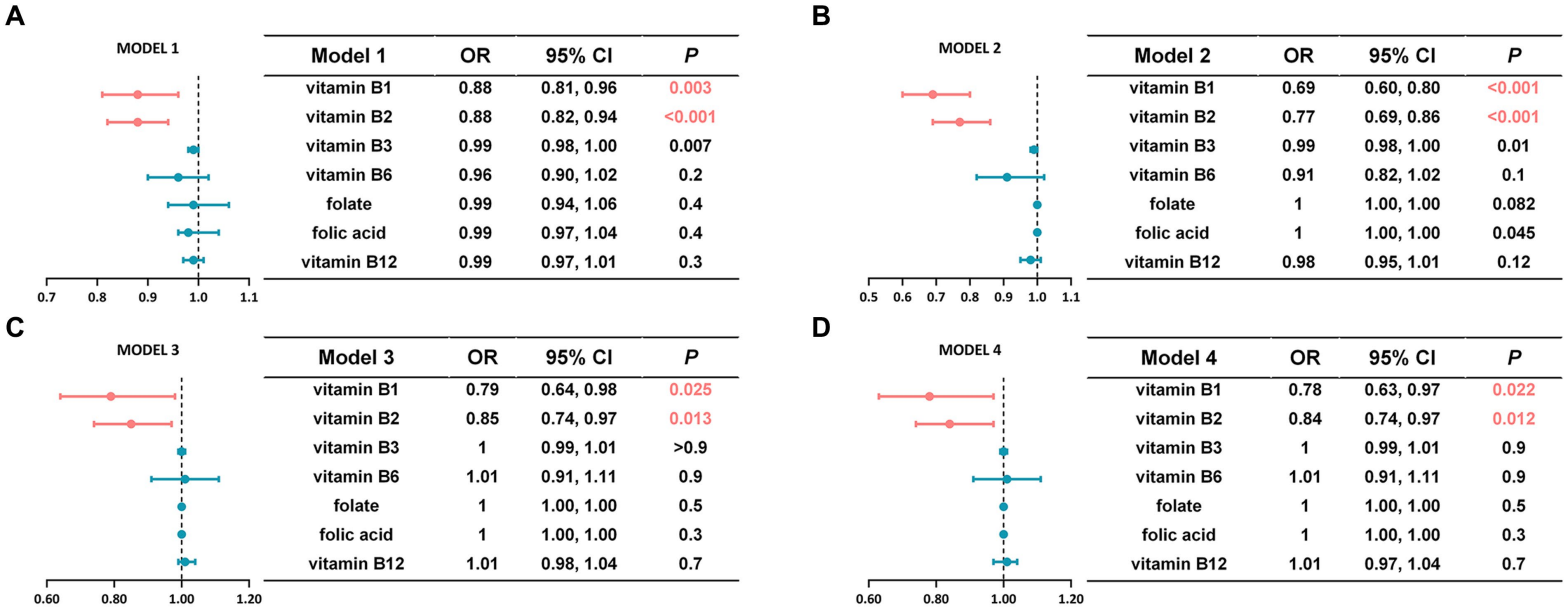
An accurate comparison and analysis of the association between B vitamins and the risk of sarcopenia under four different models. **(A)** Model 1 adjusted for gender, age, race, PIR, and dietary intake of B vitamins. **(B)** Model 2 additionally adjusted for baseline BMI, WWI, smoking, alcohol consumption, and physical activity based on Model 1. **(C)** Model 3 additionally adjusted for diseases that could lead to sarcopenia based on Model 2. **(D)** Model 4 adjusted for total dietary caloric intake, protein, carbohydrate, and fat based on Model 3. Statistically significant differences were highlighted in pink.

**Figure 3:**
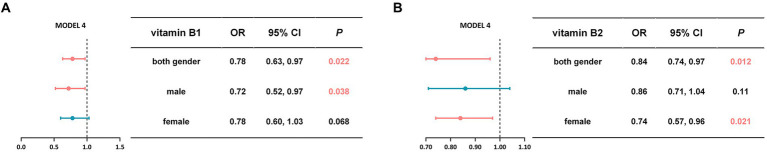
Gender-stratified subgroup analysis of the association between dietary intake of vitamins B1 and B2, and risk of sarcopenia in Model 4. **(A)** Association of vitamin B1 with the risk of sarcopenia in sex-specific populations. **(B)** Association of vitamin B2 with the risk of sarcopenia in sex-specific populations. Statistically significant differences were highlighted in pink.

Four weighted logistic regression models were employed to investigate the potential association between B vitamins and the risk of sarcopenia (Figure 2). Model 1 included demographic variables such as gender, age, race, PIR, as well as dietary intake of B vitamins. Model 2 incorporated all variables from Model 1 and adjusted for BMI, WWI, smoking, alcohol consumption, and physical activity. Model 3, which was built on Model 2, was further adjusted to account for diseases that could lead to sarcopenia, including heart failure, lung disease, diabetes, cancer, kidney disease, arthritis, and anemia. Additionally, Model 4, which was based on Model 3, included nutritional variables such as total dietary intake of calories, protein, carbohydrates, and fat. In all four models, a significant inverse association was observed between dietary intake of vitamin B1 and B2, and the risk of sarcopenia. Only demographic variables and dietary intake of B vitamins considered, each 1 mg increase in dietary vitamin B1 was associated with a 12% reduction in the risk of sarcopenia (OR: 0.88, CI: 0.81–0.96, *p* = 0.003). And each 1 mg increase in dietary vitamin B2 was also associated with a similar decrease in the risk of sarcopenia (OR: 0.88, CI: 0.82–0.94, *p* < 0.001) (Figure 2A). These associations remained robust even after confounding factors such as BMI, WWI, smoking, alcohol consumption, other chronic diseases, and nutritional variables were adjusted (Figures 2B,C). In Model 4, every 1 mg increase in dietary vitamin B1 reduced sarcopenia risk by 22% (OR = 0.78, CI = 0.63–0.97, *p* = 0.022), and every 1 mg increase in dietary vitamin B2 reduced sarcopenia risk by 16% (OR = 0.84, CI = 0.74–0.97, *p* = 0.012) (Figure 2D). Limited correlations were observed for other B vitamins. Consequently, further investigation of these vitamins was constrained due to the absence of statistically significant differences. Model 4 was selected for further analysis based on clinical significance (Table 2). In stratified analyses by gender, males showed a significant 28% reduction in the risk of sarcopenia with every 1 mg addition of dietary vitamin B1 (OR = 0.72, CI = 0.52–0.97, *p* = 0.038) (Figure 3A). Similarly, females showed a noteworthy 26% decrease in the risk of sarcopenia with every 1 mg increase in dietary vitamin B2 intake (OR = 0.74, CI = 0.57–0.96, *p* = 0.021) (Figure 3B). However, no statistically significant differences were observed between vitamin B2 intake and the male group, and between vitamin B1 and the female group within this model’s framework.

**Table 2:** Detailed information on all variables in Model 4.

Characteristic	Vitamin B1			Vitamin B2		
	OR	95% CI	*p*	OR	95% CI	*p*
**Gender**			**<0.001**			**<0.001**
Male	—	—		—	—	
Female	1.12	1.03, 1.17		1.13	1.02, 1.21	
**Age**	1.09	0.98, 1.21	0.7	1.11	0.99, 1.21	0.7
**Race**			**<0.001**			**<0.001**
Hispanic	—	—		—	—	
Non-Hispanic White	1.21	0.85, 1.72		1.24	0.87, 1.76	
Non-Hispanic Black	0.27	0.16, 0.43		0.26	0.16, 0.42	
Non-Hispanic Asian	1.25	0.89, 1.75		1.2	0.85, 1.69	
Other Race	0.92	0.47, 1.80		0.92	0.47, 1.78	
**PIR Status**			0.7			0.6
High income households	—	—		—	—	
Low income households	1.07	0.79, 1.46		1.07	0.78, 1.47	
**BMI**	0.40	0.37, 0.44	**<0.001**	0.4	0.37, 0.44	<0.001
**WWI**	1.56	1.43, 1.69	**<0.001**	1.54	1.44, 1.71	<0.001
**Alcohol usage status**			**0.007**			**0.007**
Non-drinkers	—	—	—	—		
Slight to moderate drinkers	1.39	1.20, 1.76		1.39	1.20, 1.76	
Heavy drinkers	1.58	1.39, 1.86		1.58	1.39, 1.87	
**Smoking status**			0.2			0.15
Non-smokers	—	—		—	—	
Ex-smokers	1.17	0.42, 3.27		1.21	0.44, 3.34	
Current smokers	1.26	0.96, 1.65		1.29	0.99, 1.69	
**Physical activity status**			**<0.001**			**<0.001**
Inactive group	—	—		—	—	
Moderately active group	0.77	0.46, 1.29		0.78	0.46, 1.30	
Highly active group	0.56	0.43, 0.73		0.56	0.43, 0.73	
**Congestive heart failure**			0.5			0.4
No	—	—		—	—	
Yes	0.61	0.14, 2.73		0.55	0.11, 2.67	
**Chronic lung diseases**			0.5			0.4
No	—	—		—	—	
Yes	2.17	0.23, 20.5		2.33	0.24, 22.3	
**Anemia**			0.8			0.8
No	—	—		—	—	
Yes	1.86	0.90, 2.42		1.86	0.90, 2.47	
**Arthritis**			**0.001**			**0.001**
No	—	—		—	—	
Yes	2.02	1.30, 3.16		2.06	1.31, 3.23	
**Gout**			0.6			0.5
No	—	—		—	—	
Yes	0.74	0.25, 2.21		0.72	0.24, 2.18	
**Stroke**			**0.016**			**0.028**
No	—	—		—	—	
Yes	1.22	1.06, 1.79		1.25	1.07, 1.91	
**Kidney insufficiency**			0.12			0.082
No	—	—		—	—	
Yes	1.79	0.83, 3.84		1.87	0.90, 3.88	
**Malignancies or Cancers**			0.071			0.064
No	—	—		—	—	
Yes	1.59	0.96, 5.66		1.56	0.96, 5.44	
**Diabetes**			**0.017**			**0.017**
No	—	—		—	—	
Yes	1.98	1.79, 2.42		1.94	1.79, 2.44	
**Dietary energy (kcal)**	0.81	0.78, 1.14	0.14	0.82	0.78, 1.16	0.2
**Dietary protein (g)**	0.72	0.59, 1.01	0.067	0.74	0.61, 1.03	0.077
**Dietary carbohydrate (g)**	1.04	0.99, 1.09	0.7	1.03	0.99, 1.08	0.6
**Dietary fat (g)**	1.06	0.97, 1.12	0.055	1.03	0.95, 1.09	0.074
**Dietary vitamin B1 (mg)**	0.79	0.64, 0.98	**0.025**		—	
**Dietary vitamin B2 (mg)**		—		0.85	0.74, 0.97	**0.013**

Statistical differences (*p* < 0.05) were highlighted in bold.

### Trend analysis of vitamin B1 and vitamin B2 with the risk of sarcopenia

**Figure 4:**
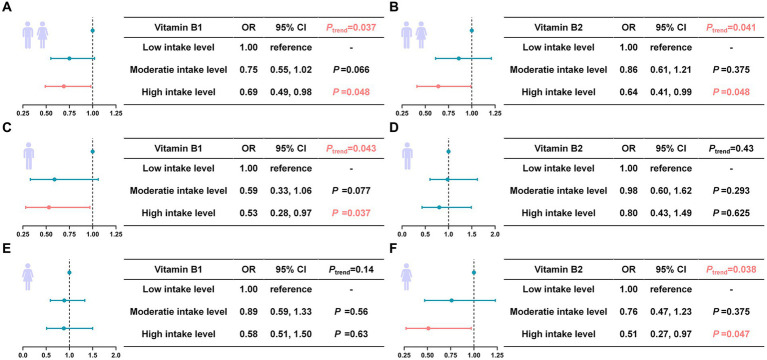
Dose–response analysis of the association between dietary intake of vitamins B1 and B2, and the risk of sarcopenia in MODEL 4. **(A, C, E)** Association between vitamin B1 intake and the risk of sarcopenia in the total, male, and female populations. **(B, D, F)** Association between vitamin B2 intake and the risk of sarcopenia in the total, male, and female populations. Statistically significant differences were highlighted in pink.

To further investigate whether there was a dose–response relationship between vitamin B1 and B2 and the risk of sarcopenia, vitamin B1 and B2 were divided into three tertiles (T1: Low intake level; T2: Moderate intake level; T3: High intake level) based on their tri-sectional quantiles intervals and included in the analysis (Figure 4). For vitamin B1, the tertiles were 0.03–1.41 mg (T1), 1.42–2.08 mg (T2), 2.09–12.05 mg (T3) for men, and 0.03–1.05 mg (T1), 1.06–1.54 mg (T2), 1.55–7.33 mg (T3) for women. For vitamin B2, the tertiles were 0.04–1.72 mg (T1), 1.73–2.57 mg (T2), 2.58–15.74 mg (T3) for men, and 0.06–1.36 mg (T1), 1.37–1.96 mg (T2), 1.97–18.53 mg (T3) for women. For vitamin B1 in total populations (Figure 4A), compared to the T1 (reference: 1), the risk of sarcopenia decreased by 25 and 31% for T2 and T3, respectively, with a significant trend (*P*_trend_ = 0.037). For vitamin B2 in total populations (Figure 4D), when comparing to T1, the risk of sarcopenia decreased by 14 and 36% for T2 and T3, respectively, with a significant trend (*P*_trend_ = 0.041). In the sex-stratified subgroup analyses, a dose-dependent association was observed between vitamin B1 intake and a reduced risk of sarcopenia in men (Figure 4B). Compared to T1, the risk of sarcopenia was reduced by 41% for T2 and 47% for the T3, with a significant trend (*P*_trend_ = 0.043). Conversely, in women, an inverse relationship was found between vitamin B2 intake and the risk of sarcopenia (Figure 4F). The risk reduction was 24% in T2 and 49% in T3, compared to T1, with a significant dose–response trend (*P*_trend_ = 0.038). And there was no statistically significance in dose–response relationship was observed in vitamin B1 for female populations (Figure 4C), and vitamin B2 for male populations (Figure 4E).

### Nonlinear associations with RCS analysis

**Figure 5:**
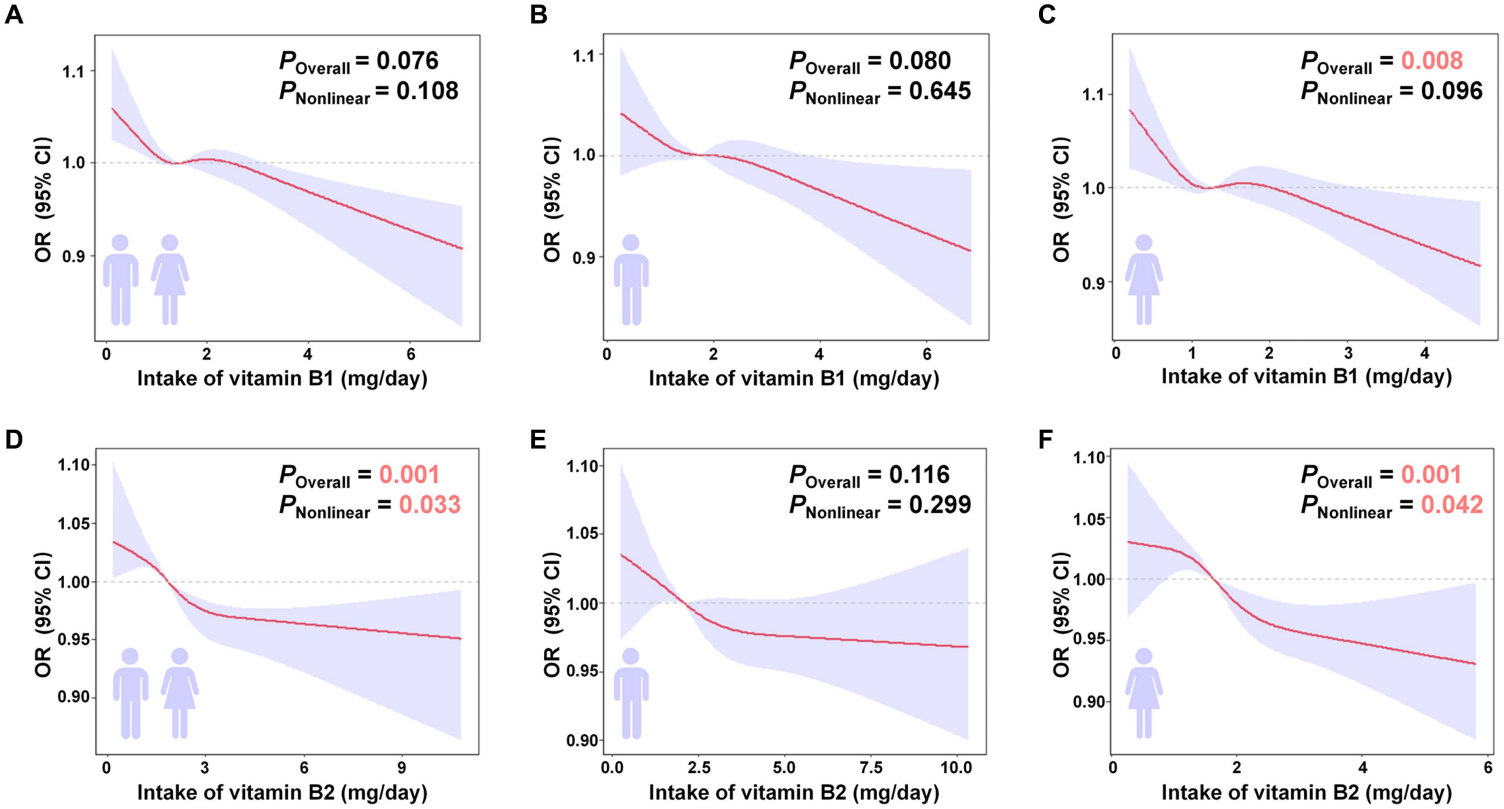
RCS analysis of the B vitamin intake and sarcopenia risk with a 4-knot restricted cubic spline model based on Model 4. **(A–C)** RCS analysis of vitamin B1 in the total, male, and female populations. **(D–F)** RCS analysis of vitamin B2 in the total, male, and female populations. Odds ratios were standardized to 1.00 for median B vitamin intake. The red line represented the trend, and the purple area was the 95% confidence interval. *P*_overall_ assessed the model’s explanatory power, and *P*_nonlinear_ signified the significance of the nonlinear association. Positive results were highlighted in pink.

The logistic regression of association between intake of vitamins B1, B2 and a lower risk of sarcopenia has been confirmed to be linear, but whether existed the nonlinear association should be explored. RCS analysis was utilized to model the intake of B vitamins and sarcopenia risk directly (Figure 5). The intake of vitamin B1 and B2 was inversely related to the risk of sarcopenia. The overall relationship between vitamin B1 intake and the risk of sarcopenia appeared to be linear, with a lack of significant non-linear trends (*P*_Overall_ = 0.076, *P*_Nonlinear_ = 0.108) (Figure 5A). In the total populations, vitamin B2 intake had both linear and nonlinear trends (*P*_Overall_ = 0.001, *P*_Nonlinear_ = 0.033) with sarcopenia risk (Figure 5D). With vitamin B2 intake of 0–3 mg/day, sarcopenia risk decreased significantly. When over 3 mg/day, the benefits become smaller but were still present. When grouped by sex, a significant nonlinear trend was observed between vitamin B2 intake and the risk of sarcopenia in female populations (*P*_Overall_ = 0.001, *P*_Nonlinear_ = 0.042) after the introduction of RCS (Figure 5F), whereas no such trend was found in males (Figure 5E). And for vitamin B1, there was no evidence of a nonlinear relationship among males (Figure 5B). Although a certain nonlinear association was observed among females in Figure 5C (*P*_Overall_ = 0.008, *P*_Nonlinear_ = 0.096), this non-linearity could not be attributed to vitamin B1 intake due to the interference of various other factors (Figure 5C).

## Discussion

This NHANES-based study utilized logistic multivariate linear regression, trend analysis, and RCS on 2011–2018 data. After adjusted for demographic, lifestyle, comorbidity, and dietary factors, an inverse association between dietary intake of vitamins B1 and B2 and the risk of early-onset sarcopenia was found, suggesting a protective effect of vitamins B1 and B2 with a significant dose–response. In contrast, other B vitamins were not significantly associated with the risk of early-onset sarcopenia. The protective effects of vitamins B1 and B2 were sex-specific: vitamin B1 reduced the risk of early-onset sarcopenia in males, and vitamin B2 reduced the risk in females.

B vitamins are involved in various aspects of energy and protein metabolism and are also linked to neurological function and integrity. Surprisingly, except for vitamins B1 and B2, other B groups showed only a subtle protective effect and no statistically significant effect in our study. This result is inconsistent with many previous reports (39, 47, 51–57). First, our data were derived from the 24-h recall of participants collected by NHANES and not directly from the detection of the food itself. This may bring some bias, as shown in Table 1. Only vitamins B1, B2, and B3 showed statistically significant differences between early-onset sarcopenia patients and controls, while other B vitamins showed no difference, suggesting that there was not enough variability in the data of other B vitamins in this study sample. Second, the active forms of B vitamins in the body are physiologically and functionally closely related, and they exert synergistic effects to some extent, participating together in the maintenance of homeostasis and balance (57–62). This synergistic effect may result in a failure to reflect the true effect of a particular B vitamin when analyzed in isolation (63). Third, other B vitamins are affected by other unconsidered confounding factors, such as genetic polymorphisms (22, 64–66), drug interference (67), bioavailability of nutrients in foods (68, 69), disease status (70–73), etc., resulting in masked or distorted relationships with the risk of early-onset sarcopenia.

Meanwhile, sex-stratified analysis found that there was a gender difference in the risk of early-onset sarcopenia. Vitamin B1 showed a more significant protective effect in males, while vitamin B2 showed a more significant protective effect in females. Our model revealed a higher risk of early-onset sarcopenia in women compared to men, consistent with previous findings (74–78). This disparity may be attributed to the influence of male androgens, which promote myogenic differentiation of mesenchymal cells, protein synthesis in muscle cells, and hypertrophy of muscle fibers (79). Additionally, sociocultural factors and the division of labor often lead to increased physical activity and manual labor among men, potentially contributing to sex-based differences in early-onset sarcopenia risk. The gender-specific impact of vitamin B1 and B2 on muscle mass is intriguing, and it could be attributed to chance or an inadequately understood mechanism. Montiel et al. demonstrated that chronic thiamine supplementation elevated serum testosterone, attenuated gonadal atrophy, and enhanced sexual activity in aged male mice, suggesting a potential interaction between vitamin B1 and androgens (80). Additionally, men generally have higher metabolic rates and are more likely to engage in alcohol consumption, which can impair vitamin B1 absorption and utilization. These factors may contribute to an increased vitamin B1 requirement in men, amplifying the protective effect of supplementation (81, 82). As for the reason why the protective effect of vitamin B2 in female population is more significant, we suspect that women need to consume large amounts of vitamin B2 during special physiological stages, such as menstruation, pregnancy, and lactation, which may lead to a relative deficiency of vitamin B2. The heightened protective effect of vitamin B2 in females may stem from increased demand during specific physiological stages like menstruation, pregnancy, and lactation, potentially resulting in relative deficiency (51). Limited research indicated a correlation between vitamin B2 deficiency and conditions such as diabetes (83), periodontitis (84), cataracts (85), and depression (86), with a stronger association in females. These observations warrant further investigation into the gender-specific mechanisms underlying the effects of vitamin B2.

Additionally, RCS modeling showed a threshold effect of vitamin B2 intake on early-onset sarcopenia risk, with a protective effect up to 3 mg/d, unlike vitamin B1. This might be related to the different absorption, metabolism, and roles of vitamins B1 and B2 in energy pathways and muscle function. Vitamin B1 absorption and utilization may be high, resulting in similar vitamin B1 levels at different intakes. Vitamin B2 absorption and utilization may be low, and excessive intake may increase kidney excretion. Vitamin B1 mainly participates in glucose metabolism, while vitamin B2 participates in fatty acid and amino acid metabolism. Muscle, as an organ with high metabolic capacity, may require more vitamin B1 but less vitamin B2.

The study focused on dietary B vitamins intake, as supplements and total intakes (diet plus supplements) showed no significant differences. Therefore, supplement data were not reported. Supplements use may depend on factors such as income, education, health status, awareness, and advice from health workers. These factors may also lower sarcopenia risk, making supplement data less reflective of the relationship between B vitamin intake and sarcopenia risk. Further information on the use of dietary supplements was available in Supplementary Table S2.

This study showed that sarcopenia risk was highest in non-Hispanic Asians and lowest in non-Hispanic blacks, in line with previous studies (87–90). People of different ethnicities may be affected by different genetics, environment, lifestyle, nutrition, and diseases, which may influence changes in muscle mass and function (91). Genetics, environment, lifestyle, nutrition, and diseases may affect muscle mass and function in different ethnicities. For instance, some studies found that African descent people had higher muscle power and endurance, while Asian descent people were influenced by social and economic factors and had lower physical activity levels (92, 93). However, current research is limited. Moreover, obesity indicators had conflicting results, with BMI negatively associated with sarcopenia and WWI positively associated. Both measures assessed obesity, but reflected different body composition. BMI did not differentiate muscle and fat mass and distribution, whereas WWI indicated abdominal fat accumulation. Studies confirmed that abdominal fat was harmful and linked to metabolic syndrome, inflammation, and sarcopenia (94–96). BMI and WWI may not be independent factors but influence each other. There may be a U-shaped relationship between BMI and WWI, such that people with lower or higher BMI have higher WWI and those with moderate BMI have lower WWI (97, 98). Furthermore, the study found that alcohol use increased sarcopenia risk, while physical exercise reduced it, consistent with previous researches (4, 99–105). Among chronic comorbidities, stroke, diabetes, and arthritis increased sarcopenia risk, possibly due to inflammatory immune response worsening muscle mass loss in affected individuals (8, 106–111). Other chronic diseases had no significant associations, so we speculated that their co-occurrence may have masked the effect of certain conditions when analyzed separately. Finally, surprisingly, our model showed no significant association between protein intake and early-onset sarcopenia, which can be attributed to a chance (Table 2).

The study also has some limitations. Firstly, it is a cross-sectional study, which restricts the establishment of causal relationships between B vitamins intake and sarcopenia risk, allowing only associative patterns. Secondly, due to the absence of a universally accepted diagnosis for sarcopenia, encompassing both muscle mass loss and muscle function decline, the lack of data on 20-foot timed walk and grip strength tests in NHANES could introduce bias. Thirdly, similar to other large-scale epidemiological studies, the NHANES questionnaire and 24-h recall method may be influenced by self-report bias, despite the implementation of the AMPM.

Despite these limitations, the study holds clinical and public health significance. NHANES is the sole national survey in the US that evaluates the comprehensive nutritional intake of food, beverages, and supplements in a diverse and representative population of noninstitutionalized Americans, providing a large and robust sample size for the study. Furthermore, this research was the first to investigate the relationship between B vitamins and sarcopenia in non-senile patients, even in a younger age group. Additionally, the study revealed a negative association between vitamin B1 and B2 intake and sarcopenia, suggesting a novel approach for potential preventive and therapeutic interventions. And further research is needed to validate the preventive and therapeutic effects of vitamins B1 and B2 on early-onset sarcopenia.

## Conclusion

In our study, we utilized multiple logistic regression models and RCS to investigate the association between vitamin B1 and B2 intake and the risk of early-onset sarcopenia. Our findings revealed a significant correlation, indicating that higher intake of these vitamins is linked to a reduced risk of developing sarcopenia. Notably, our sex-stratified subgroup analysis demonstrated a sex-specific effect, with vitamin B1 exhibiting stronger protective effects in males while vitamin B2 showed greater benefits in females. These gender disparities highlighted the importance of considering sex differences when addressing preventive measures for sarcopenia. Last but not the least, we call on researchers to further explore the relationship between B vitamins and sarcopenia among young individuals, as well as delve into potential underlying mechanisms based on our study’s outcomes.

## Data availability statement

The original contributions presented in the study are included in the article/Supplementary materials, further inquiries can be directed to the corresponding authors.

## Ethics statement

The studies involving humans were approved by Ethics Review Board of the National Center for Health Statistics at the CDC. The studies were conducted in accordance with the local legislation and institutional requirements. Written informed consent for participation was not required from the participants or the participants’ legal guardians/next of kin because NHANES is approved by the Ethics Review Board of the National Center for Health Statistics at the CDC, and no additional ethical review is required for the use of NHANES open-source data. Written informed consent was not obtained from the individual(s) for the publication of any potentially identifiable images or data included in this article.

## Author contributions

SY: Conceptualization, Data curation, Writing – original draft, Writing – review & editing. ZD: Formal analysis, Software, Writing – original draft, Writing – review & editing. JZ: Formal analysis, Investigation, Supervision, Writing – original draft. LY: Data curation, Investigation, Writing – original draft. YX: Methodology, Project administration, Validation, Writing – review & editing. XL: Resources, Visualization, Writing – original draft. ZZ: Conceptualization, Data curation, Investigation, Writing – original draft. XK: Methodology, Validation, Writing – original draft. KT: Supervision, Validation, Writing – review & editing. MC: Funding acquisition, Writing – review & editing, Conceptualization, Supervision. LF: Data curation, Conceptualization, Writing – review & editing.
