# Supplementary material for: Association of vitamins B1 and B2 intake with early-onset sarcopenia in the general adult population of the US: a cross-sectional study of NHANES data from 2011 to 2018
Source: Front Nutr. 2024 Mar 8;11:1369331. doi: 10.3389/fnut.2024.1369331 (PMC10976947; doi:10.3389/fnut.2024.1369331)
Supplement: Supplementary file 1 [file Data_Sheet_1.docx]

**Supporting Files**

**Association of Vitamins B1 and B2 Intake with Early-Onset Sarcopenia in the General Adult Population of the US: a Cross-sectional Study of NHANES Data from 2011 to 2018**

**Sha Yang^1#^, Zhenyu Dong^2#^, Jiaqi Zhao^3#^, Lijia Yuan^4^,** **Yao Xiao^4^, Xing Luo^1^, Zhuyang Zhao^1^, Xia Kang^5^, Kanglai Tang^2*^, Ming Chen^1*^, Lie Feng^1*^**

1. Department of Clinical Laboratory Medicine, Southwest Hospital, Third Military Medical University, Chongqing, China

2. Department of Orthopeadics/Sports Medicine Center, Key Laboratory of Sports Injury Repair and Reconstruction, Southwest Hospital, Third Military Medical University, Chongqing, China

3. Department of Orthopedic Surgery, Southwest Hospital, Third Military Medical University, Chongqing, China

4. Emergency department, Southwest Hospital, Third Military Medical University, Chongqing, China

5.Pancreatic Injury and Repair Key Laboratory of Sichuan Province, The General Hospital of Western Theater Command, Chengdu, Sichuan, China.

**Corresponding Author**

* Liu Feng

Email: 275290568@qq.com

* Ming Chen

Email: [chming1971@126.com](mailto:chming1971@126.com)

* Kanglai Tang

Email: tangkanglai@hotmail.com

^#^ Sha Yang, Zhenyu Dong and Jiaqi Zhao contributed equally to this work.

**Table S1. Baseline characteristics of total participants**

| **Characteristic** | **Non-sarcopenia participants**  **(N=7523)** | **sarcopenia participants**  **(N=1188)** | ***P*** |
| --- | --- | --- | --- |
| **Gender** |  |  | 0.3 |
| Male | 3,776 (51%) | 617 (52%) |  |
| Female | 3,747 (49%) | 571 (48%) |  |
| **Age** | 39 (±12) | 38 (±13) | **0.007** |
| **Race** |  |  | **<0.001** |
| Hispanic | 1,908 (17%) | 207 (12%) |  |
| Non-Hispanic White | 2,761 (63%) | 483 (68%) |  |
| Non-Hispanic Black | 1,672 (12%) | 92 (4.2%) |  |
| Non-Hispanic Asian | 852 (4.5%) | 361 (12%) |  |
| Other Race | 330 (3.7%) | 45 (3.7%) |  |
| **PIR Status** |  |  | 0.15 |
| high-income households | 5,148 (78%) | 805 (75%) |  |
| low-income households | 2,375 (22%) | 383 (25%) |  |
| **BMI** | 30 (±6) | 22 (±3) | **<0.001** |
| **WWI** | 10.83 (±0.78) | 10.49 (0.68) | **<0.001** |
| **Alcohol Usage Status** |  |  | 0.091 |
| non-drinkers | 871 (8.8%) | 189 (11%) |  |
| slight to moderate drinkers | 5,982 (80%) | 897 (78%) |  |
| heavy drinkers | 670 (11%) | 102 (11%) |  |
| **Smoking Status** |  |  | 0.1 |
| non-smokers | 4,535 (60%) | 713 (54%) |  |
| ex-smokers | 141 (2.1%) | 13 (1.9%) |  |
| current smokers | 2,847 (38%) | 462 (44%) |  |
| **Physical Activity Status** |  |  | 0.2 |
| Inactive group | 3,625 (48%) | 640 (51%) |  |
| moderately active group | 629 (9.0%) | 111 (9.7%) |  |
| highly active group | 3,269 (43%) | 437 (39%) |  |
| **Malignancies or Cancers** |  |  | 0.15 |
| No | 7,233 (95%) | 1,139 (94%) |  |
| Yes | 290 (4.8%) | 49 (6.4%) |  |
| **Chronic Lung Diseases** |  |  | **0.009** |
| No | 7,203 (95%) | 1,119 (92%) |  |
| Yes | 320 (5.1%) | 69 (8.0%) |  |
| **Diabetes** |  |  | **<0.001** |
| No | 6,914 (92%) | 1,119 (90%) |  |
| Yes | 609 (8.1%) | 113 (9.5%) |  |
| **Congestive Heart Failure** |  |  | 0.058 |
| No | 7,451 (99%) | 1,180 (100%) |  |
| Yes | 72 (0.8%) | 8 (0.4%) |  |
| **Anemia** |  |  | 0.9 |
| No | 7,260 (97%) | 1,156 (97%) |  |
| Yes | 263 (3.0%) | 32 (2.8%) |  |
| **Arthritis** |  |  | 0.8 |
| No | 6,481 (86%) | 1,046 (85%) |  |
| Yes | 1,042 (14%) | 142 (15%) |  |
| **Gout** |  |  | **0.011** |
| No | 7,334 (97%) | 1,172 (99%) |  |
| Yes | 189 (2.5%) | 16 (1.0%) |  |
| **Stroke** |  |  | 0.4 |
| No | 7,417 (99%) | 1,178 (99%) |  |
| Yes | 106 (1.1%) | 10 (0.7%) |  |
| **Kidneys Insufficiency** |  |  | 0.077 |
| No | 7,401 (98%) | 1,158 (98%) |  |
| Yes | 122 (1.6%) | 30 (2.5%) |  |
| **Dietary Energy (kcal)** | 2,064 (1,611, 2,631) | 2,003 (1,522, 2,560) | **0.007** |
| **Supplements Energy (kcal)** | 15 (10, 23) | 13 (10, 20) | 0.4 |
| **Total Energy (kcal)** | 2,068 (1,614, 2,639) | 2,005 (1,522, 2,560) | **0.006** |
| **Dietary Protein(g)** | 81 (60, 104) | 76 (55, 99) | **<0.001** |
| **Supplements Protein(g)** | 1.0 (0.5, 1.5) | 0.5 (0.5, 1.0) | **0.009** |
| **Total Protein(g)** | 81 (60, 104) | 76 (55, 99) | **<0.001** |
| **Dietary Carbohydrate (g)** | 240 (180, 312) | 240 (178, 320) | 0.8 |
| **Supplements Carbohydrate (g)** | 3.0 (1.0, 4.0) | 3.0 (1.0, 4.0) | 0.6 |
| **Total Carbohydrate (g)** | 241 (181, 312) | 240 (178, 320) | 0.8 |
| **Dietary Fat (g)** | 79 (58, 105) | 74 (56, 98) | **<0.001** |
| **Supplements Fat (g)** | 1.25 (1.00, 2.00) | 1.00 (1.00, 2.00) | 0.6 |
| **Total Fat (g)** | 79 (58, 106) | 74 (56, 99) | **<0.001** |
| **Dietary Vitamin B1 (mg)** | 1.50 (1.11, 2.01) | 1.49 (1.08, 1.97) | **0.047** |
| **Supplements Vitamin B1 (mg)** | 2 (2, 6) | 2 (2, 5) | >0.9 |
| **Total Vitamin B1 (mg)** | 1.7 (1.2, 2.7) | 1.7 (1.2, 2.5) | 0.15 |
| **Dietary Vitamin B2 (mg)** | 1.98 (1.45, 2.67) | 1.86 (1.37, 2.49) | **0.002** |
| **Supplements Vitamin B2 (mg)** | 2 (2, 5) | 2 (2, 3) | 0.4 |
| **Total Vitamin B2 (mg)** | 2.2 (1.6, 3.5) | 2.1 (1.5, 3.3) | **0.009** |
| **Dietary** **Vitamin B3 (mg)** | 25 (18, 33) | 23 (17, 31) | **0.005** |
| **Supplements Vitamin B3 (mg)** | 20 (16, 25) | 20 (14, 20) | 0.3 |
| **Total Vitamin B3 (mg)** | 29 (20, 41) | 27 (19, 39) | **0.029** |
| **Dietary Vitamin B6 (mg)** | 1.92 (1.37, 2.63) | 1.84 (1.34, 2.59) | 0.12 |
| **Supplements Vitamin B6 (mg)** | 3 (2, 6) | 3 (2, 6) | 0.3 |
| **Total Vitamin B6 (mg)** | 2.3 (1.5, 3.9) | 2.2 (1.5, 3.9) | 0.3 |
| **Dietary Folate (μg)** | 143 (84, 232) | 143 (83, 246) | 0.5 |
| **Supplements Folate (μg)** | 400 (400, 400) | 400 (400, 400) | 0.4 |
| **Total Folate (μg)** | 191 (101, 442) | 196 (105, 440) | >0.9 |
| **Dietary Folic acid (μg)** | 465 (332, 661) | 474 (325, 674) | 0.7 |
| **Supplements Folic acid (μg)** | 680 (680, 680) | 680 (680, 680) | 0.4 |
| **Total Folic acid (μg)** | 569 (371, 980) | 574 (368, 982) | 0.9 |
| **Dietary Vitamin B12 (μg)** | 4.1 (2.6, 6.2) | 3.8 (2.5, 6.0) | 0.3 |
| **Supplements Vitamin B12 (μg)** | 18 (6, 50) | 18 (6, 74) | 0.053 |
| **Total Vitamin B12 (μg)** | 5 (3, 11) | 5 (3, 11) | 0.7 |
| **Dietary Vitamin B1 (tertiles)** |  |  | 0.4 |
| Low intake level | 2493 (33.7%) | 409 (35.7%) |  |
| Moderate intake level | 2516 (33.2%) | 389 (32.3%) |  |
| High intake level | 2514 (32.1%) | 390 (32.0%) |  |
| **Dietary Vitamin B2 (tertiles)** |  |  | **0.031** |
| Low intake level | 2503 (32.9%) | 403 (36.4%) |  |
| Moderate intake level | 2500 (33.1%) | 404 (35.2%) |  |
| High intake level | 2540 (34.0%) | 361 (28.4%) |  |
| **SMI** | 8.21 (6.98, 9.28) | 6.08 (5.27, 7.04) | **<0.001** |

Note: Age, BMI, and WWI exhibit Gaussian distribution (mean ± SD); nutritional variables and SMI deviate (IQR: P50 (P25, P75)); categorical variables were denoted as unweighted n (weighted %). Categorical and continuous variables were compared via chi-squared and Wilcoxon rank-sum tests with Rao & Scott’s correction for complex surveys. Statistical differences (*P*<0.05) were highlighted in bold.

**Table S2. Detailed information on the use of dietary supplements**

| **Characteristic** | **Non-sarcopenia participants**  **N=7523** | **sarcopenia participants**  **N=1188** | ***P*** |
| --- | --- | --- | --- |
| **Supplements Energy** |  |  | 0.5 |
| Not use | 6,286 (81%) | 1,004 (82%) |  |
| Use | 1,237 (19%) | 184 (18%) |  |
| **Supplements Protein** |  |  | 0.057 |
| Not use | 7,328 (97%) | 1,167 (98%) |  |
| Use | 195 (3.2%) | 21 (1.7%) |  |
| **Supplements Carbohydrate** |  |  | 0.5 |
| Not use | 6,749 (88%) | 1,064 (89%) |  |
| Use | 774 (12%) | 124 (11%) |  |
| **Supplements Fat** |  |  | 0.5 |
| Not use | 6,880 (90%) | 1,099 (91%) |  |
| Use | 643 (9.6%) | 89 (8.6%) |  |
| **Supplements Vitamin B1** |  |  | 0.3 |
| Not use | 6,076 (78%) | 956 (80%) |  |
| Use | 1,447 (22%) | 232 (20%) |  |
| **Supplements Vitamin B2** |  |  | 0.2 |
| Not use | 6,071 (78%) | 954 (80%) |  |
| Use | 1,452 (22%) | 234 (20%) |  |
| **Supplements Vitamin B3** |  |  | 0.9 |
| Not use | 5,938 (76%) | 922 (76%) |  |
| Use | 1,585 (24%) | 266 (24%) |  |
| **Supplements Vitamin B6** |  |  | 0.5 |
| Not use | 5,826 (74%) | 901 (76%) |  |
| Use | 1,697 (26%) | 287 (24%) |  |
| **Supplements Folate** |  |  | 0.5 |
| Not use | 5,846 (75%) | 907 (76%) |  |
| Use | 1,677 (25%) | 281 (24%) |  |
| **Supplements Folic acid** |  |  | 0.5 |
| Not use | 5,846 (75%) | 907 (76%) |  |
| Use | 1,677 (25%) | 281 (24%) |  |
| **Supplements Vitamin B12** |  |  | >0.9 |
| Not use | 5,737 (73%) | 893 (73%) |  |
| Use | 1,786 (27%) | 295 (27%) |  |

Note: The calorie, carbohydrates, protein, fats, and various types of vitamin B present in the dietary supplements were displayed separately as categorical variables in the form of unweighted n (weighted %). Chi-squared tests were used to statistically analyze the differences between the two groups.
